# Lymphadenopathy in the rheumatology practice: a pragmatic approach

**DOI:** 10.1093/rheumatology/kead644

**Published:** 2023-12-18

**Authors:** Stefano Rodolfi, Emanuel Della-Torre, Lucia Bongiovanni, Puja Mehta, David C Fajgenbaum, Carlo Selmi

**Affiliations:** Department of Rheumatology and Clinical Immunology, IRCCS Humanitas Research Hospital, Milan, Italy; Department of Biomedical Sciences, Humanitas University, Milan, Italy; Università Vita-Salute San Raffaele, IRCCS San Raffaele Scientific Institute, Milan, Italy; Unit of Immunology, Rheumatology, Allergy and Rare Diseases (UnIRAR), Milan, Italy; IRCCS San Raffaele Scientific Institute, Milan, Italy; Università Vita-Salute San Raffaele, IRCCS San Raffaele Scientific Institute, Milan, Italy; Department of Haematopathology Diagnostic Area, Unit of Pathology, IRCCS San Raffaele Scientific Institute, Milan, Italy; Division of Medicine, University College, Centre for Inflammation and Tissue Repair, UCL Respiratory, London; Department of Rheumatology, University College London Hospital (UCLH), London, UK; Department of Medicine, Division of Translational Medicine and Human Genetics, Center for Cytokine Storm Treatment and Laboratory, Philadelphia, PA, USA; Department of Rheumatology and Clinical Immunology, IRCCS Humanitas Research Hospital, Milan, Italy; Department of Biomedical Sciences, Humanitas University, Milan, Italy

**Keywords:** lymph node enlargement, arthritis, connective tissue disease, sarcoidosis, IgG4-related disease, Castleman disease, siltuximab

## Abstract

Lymphadenopathy is a common clinical finding and diagnostic challenge within general medicine and rheumatology practice. It may represent a primary manifestation of an underlying immune-mediated disease or indicate an infectious or neoplastic complication requiring differing management. Evaluating lymphadenopathy is of particular relevance in rheumatology, given that lymph node enlargement is a common finding within the clinical spectrum of several well-known rheumatologic disorders including RA, SLE and SS. In addition, lymphadenopathy represents a hallmark manifestation of rare immunological diseases such as Castleman disease and IgG4-related disease that must be considered in the differential diagnosis because effective targeted treatments can now impact the prognosis of these conditions. In this review we present an overview of the clinical significance of lymphadenopathy in common and rare rheumatologic diseases and propose a practical approach to lymphadenopathy in the rheumatology practice. Differential diagnosis of Castleman disease and therapeutic options for this condition of increasing rheumatologic interest will be discussed in detail.

Rheumatology key messages

- Lymph node enlargement is common in rheumatological and autoimmune diseases, frequently associated with disease activity.
- Lymphadenopathy observed in the rheumatology practice should always consider malignancy and infection as the primary causes.
- Rare diseases such as IgG4-related disease and Castleman disease must be considered in the differential diagnosis.

## The general approach to lymphadenopathy

Lymphadenopathy represents a major diagnostic challenge, because it may be observed in a variety of neoplastic, infectious and inflammatory diseases. The differential diagnosis of lymphadenopathy in rheumatologic disorders is not straightforward: it may be the consequence of a primary immune activation and a benign aspect of the natural history of the immune-mediated condition, represent an infectious complication of immunosuppressive therapies, correlate to an unrelated immune process (i.e. vaccination), or indicate a lymphoproliferative transformation or metastasis from distant solid cancers requiring immediate management.

History should focus on the presence of localized signs or symptoms that may hint towards infection or malignancy (e.g. presence of a wound or a suspicious nodule, respectively), as well as on potential exposures associated with infection (e.g. contact with animals, undercooked food, risk factors). The presence of constitutional symptoms such as low-grade fever (i.e. <38°C), night sweats and unexplained weight loss are not differentiating. In contrast, high fever (i.e. >38°C) is most commonly associated with infections. An accurate drug history is also helpful because of medications potentially causing serum sickness [1–4]. Finally, acute onset of lymph node enlargement is more suggestive of an infectious and iatrogenic cause, whilst subacute or insidious onset points towards malignant and inflammatory causes.

### Physical examination

Localized swelling typically points towards an infectious condition in the area of node drainage, even though some systemic diseases [e.g. certain lymphomas and unicentric Castleman disease (CD)] may present with localized lymphadenopathy [5–7]. On the other hand, generalized lymphadenopathy is associated with lymphoproliferative disorders and systemic rheumatologic conditions [8]. Of note, supraclavicular or axillary lymphadenopathy should raise particular attention, because they are frequent sites of tumour metastasis [9, 10]. The most important characteristics to evaluate during physical examination of enlarged lymph nodes are size, consistency, fixation and tenderness. ‘Red flags’ for malignancy are a diameter >1cm (2 cm for inguinal lymph nodes), hard consistency, fixation to subcutaneous tissue and absence of tenderness, even though these characteristics can also be found in inflammatory non-neoplastic diseases such as CD and IgG4-related disease (IgG4-RD) [7, 10].

### Imaging

Doppler US is an easily accessible tool with which to investigate lymphadenopathy. The presence of nodal matting, surrounding soft tissue oedema and prominent hilar vascularity, for instance, are suggestive of a reactive inflammatory process. Features suggestive of underlying malignancy are nodal hypoechogenenicity and alteration in nodal vascularization, with presence of avascular areas, displacement of vessels and peripheral vascularity.

CT and MRI techniques allow better characterization of nodal tissue, assessment of distribution and possible identification of other pathological lesions.^18^Fluorodeoxyglucose (18F-FDG) PET is often able to discriminate between reactive and malignant lymph nodes based on the levels of standardized uptake values (SUVs) and selecting the most active lymph node for diagnostic biopsy. US and CT also represent indispensable tools to guide lymph node biopsy or fine-needle aspiration. Table 1 illustrates the most important clinical and radiological ‘red flags’ that may point towards a neoplastic or infectious aetiology of lymphadenopathy.

**Table 1.** ‘Red flags’ associated with malignancy or infection that may be useful in the differential diagnosis of lymphadenopathy

Concern for malignancy			
Features on physical examination	Localization	Sonographic features	Associated symptoms
Firm nodule	Supraclavicular lymph nodes	Nodal hypoechogenicity	Low-grade fever
Rapid increase in size	Axillary lymph nodes in women	Presence of avascular areas	Unexplained weight loss
Fixation to s.c. tissue		Displacement of vessels	Generalized pruritus
Size >1 cm (2 cm for inguinal lymph nodes)		Peripheral vascularity	
Absence of tenderness			
Concern for infection			
Clinical history	Localization	Associated symptoms	
Acute onset	Proximity to a wound	Recent-onset high-grade fever	
History of i.v. drug abuse			
History of high-risk sexual behaviour			
Cat scratch			
Recent travel to tropical areas			

### Histological findings

Open excisional biopsy allows the histologic evaluation of intact tissue, providing information about the cellularity and architecture, and is preferred when lymphoma is suspected. While core needle biopsy provides limited information regarding lymph node architecture, it bears lower morbidity and provides some information on tissue architecture and material for immunohistochemical, genetic and molecular analysis. Complete lymph node excision is generally preferred but core biopsy is recommended when anatomical accessibility precludes excising an intact lymph node. Lastly, fine-needle aspiration provides cells for cytological analysis, most useful in cases of suspected recurrence of malignancy. Lack of information about tissue architecture and limited sampling renders this technique minimally useful for the diagnosis of lymphoma.

## Rheumatologic diseases with lymphadenopathy

### Systemic lupus erythematosus

Epidemiological data reported a prevalence of lymphadenopathy in SLE patients ranging from 33% to 69% [11–13]. Enlarged lymph nodes in SLE tend to be soft and non-tender, more often with a diameter <1 cm [11], which is below the threshold for being considered enlarged by many physicians. Lymphadenopathy is more frequently reported as generalized, while localized lymphadenopathy has been described in up to 25% of cases [11–13] and is most frequently observed in younger patients, in the context of an active disease [11]. Lymphadenopathy in SLE patients has indeed been correlated with higher frequency of constitutional symptoms, cutaneous involvement, hepatosplenomegaly, increased titers of anti-dsDNA antibodies and lower complement levels [11]. Moreover, lymphadenopathy tends to resolve with steroid therapy [13]. Although lymphadenopathy may rarely represent the initial presenting sign of SLE, it is not included in SLE classification criteria [14–17]. Increased lymph node 18F-FDG uptake at PET/CT has been described in few SLE cases, however its interpretation represents a significant challenge as it potentially mimicks lymphoma [18, 19]. The histological findings in lymph node specimens are usually nonspecific and consist of follicular hyperplasia associated with increased vascularity and scattered immunoblasts and plasma cells [20]. The most specific but variably present histological finding is coagulative necrosis with haematoxylin bodies (amorphous aggregates of periodic acid–Schiff-positive basophilic material) or reactive follicular hyperplasia; this presentation is rarely seen [20]. Some lesions may be similar to the hyaline-vascular or mixed histopathological subtypes of CD [21]. Given the clinical overlap between SLE and multicentric CD (MCD), having overlapping histopathological features presents a challenge to making an accurate diagnosis. Similarly, an important differential diagnosis is Kikuchi-Fujimoto disease, another condition with histological presentation highly reminiscent of SLE that can be found in association with SLE [22–24].

### Rheumatoid arthritis

Lymphadenopathy is a frequent finding in the clinical course of RA and has been reported in up to 82% of patients [13, 24]. Lymphadenopathy in RA tends to be predominantly localized, with preferential involvement of lymph nodes near joints with active arthritis, but swollen lymph nodes tend to be larger than in SLE, with a diameter commonly >1cm [13]. This anatomical localization likely reflects the role of lymph nodes and lymphatics in the pathophysiology of inflammatory erosive arthritis [25, 26]. Albeit infrequent, generalized lymphadenopathy with constitutional symptoms has been described at the onset of RA [27]. Not surprisingly, the most affected region is the axilla, as it drains afferent lymphatics from the upper limbs joints [13, 28]. Presence of lymphadenopathy has been correlated with both local and systemic disease activity, specifically correlating with number of tender and swollen joints, higher CRP levels, higher Simple Disease Activity Index, and higher frequency of RA-related interstitial lung disease [29, 30]. Moreover, alterations of sonographic pattern at Power Doppler signal of axillary lymph nodes were found to correlate with US synovitis degree of affected joints [31]. The role of 18F-FDG-PET, despite being useful in monitoring disease severity and treatment response in RA [32], is not established in the assessment of lymphadenopathy in RA, as increased tracer uptake may mimic lymphoma [33]. Similar to what is reported in SLE, lymphadenopathy tends to be responsive to anti-inflammatory treatment with systemic glucocorticoids, with improvement correlating with remission of synovitis [13]. The response to anti-TNF treatment assessed clinically and at synovial US was demonstrated to correlate with improvement in lymph node Power Doppler scores. Interestingly, the subset of patients with a lower number of lymph nodes involved and lower perfusion scores at baseline despite active arthritis significantly correlate with poorer response to TNF blockade [31]. At histology, RA lymphadenopathy is characterized by marked follicular hyperplasia with possible neutrophilic infiltrate in sinuses and interfollicular areas. After immunosuppressive therapy, follicular hyperplasia regresses and interfollicular and paracortical areas become hyperplastic [34]. Although this is not included in diagnostic guidelines, presence of lymphadenopathy may be an additional tool to differentiate between RA and PsA, as it is significantly more frequent in the former [35].

### Sjögren’s syndrome

SS represents one of the rheumatologic conditions in which lymphadenopathy is most commonly observed [36]. Benign, reactive lymphadenopathy is observed in 10–56% of cases [37–39], with patients tending to be younger and with a shorter disease duration, and with a predominance of females over males [37–39]. Over 90% of cases are localized with involvement of cervical lymph nodes, while other possible involved areas are the axillary and supraclavicular regions [38]. Extra-glandular involvement is significantly more common in patients with lymphadenopathy [38, 39]. More specifically, the frequency of salivary gland swelling, peripheral nervous system involvement, palpable purpura, glomerulonephritis, neutropenia and lymphopenia is higher in patients with lymphadenopathy [38, 39]. Furthermore, positivity rates of ANA and anti-Ro-SSA antibodies are higher in this subset of patients, while correlation with higher titres of RFs and cryoglobulinemia has been reported but not confirmed in different cohorts [38, 39]. Finally, lymphadenopathy has been reported to correlate with higher focus score [38]. Overall, lymphadenopathy correlates with disease activity and this has been formalized with the inclusion of lymphadenopathy in the EULAR Sjögren’s Syndrome Activity Index (ESSAI) [40]. All these features correlate with an increased risk of lymphoma development. Indeed, lymphadenopathy has been indicated as a potential predictor of lymphoma development [41–43]. Similar to SLE and RA, benign lymphadenopathy in SS seems to be characterized by reactive follicular hyperplasia with increased number of lymphoid follicles, similar to that observed in the ectopic lymphoid tissue of SS salivary glands [36, 38].

### Sarcoidosis

Despite the fact that sarcoidosis may affect any organ, lymphadenopathy is the most frequent clinical manifestation [44], with hilar lymphadenopathy seen in >90% of cases [45] and peripheral lymphadenopathy observed in 20–45% of cases [46]. Lymph nodes tend to be moderately swollen but painless, with preferential bilateral involvement of the cervical, axillary, inguinal and epitrochlear stations [45, 46]. A recent retrospective multicentric study documented hilar lymphadenopathy significantly less frequently in patients with neurological, gastrointestinal and nephrological involvement. Peripheral lymphadenopathy instead was significantly more common in patients with concomitant hepatosplenic involvement and bone involvement [45]. Histological presentation most commonly consists of non-necrotizing granulomas [46], formed by epithelioid histiocytes cells with scattered small lymphocytes and Langherans-type giant cells, while only 10% of cases may present with some small areas of fibrinoid necrosis [34]. Histology is often obtained from intrathoracic lymph nodes, requiring execution of endobronchial US (EBUS)-based techniques [47]. Despite not being sufficient to formulate a diagnosis, some sonographic features at EBUS have been found to be predictive of sarcoidosis, including the absence of coagulation necrosis, the presence of nodal conglomeration and the presence of the septal vessel sign in the colour Doppler mode [48]. Needle sampling of cervical lymph nodes identified by neck US is emerging as a potential alternative diagnostic tool when EBUS is poorly tolerated or difficult to access [49, 50]. The differential diagnosis is broad, but most importantly requires ruling out the presence of lymphoma, lung cancer, and granulomatous lymphadenitis from mycobacterial or fungal organisms [34]. Both malignancy and sarcoidosis display high 18F-FDG uptake, thereby 18F-FDG-PET/CT may be useful to select the most appropriate biopsy site but is not able to differentiate the two entities [46]. Table 2 gives an overview of the characteristics of lymphadenopathy in common rheumatological diseases.

**Table 2.** Overview on the clinical and histopathological characteristics of lymphadenopathy in common rheumatological diseases

	SLE	RA	Primary SS	Sarcoidosis
Lymphadenopathy (%)	33–69	50–80	10–56	>90 (hilar) 20–45 (peripheral)
Lymphadenopathy type	Generalized	Localized	Localized	Generalized
Preferential lymph node station	Cervical	Axillary	Cervical	Hilar
Histology	Follicular hyperplasia with coagulative necrosis and haematoxylin bodies	Follicular hyperplasia with possible neutrophilic infiltrate	Follicular hyperplasia	Non-necrotizing non-caseating granulommas
Correlation with disease activity	+	+	+	+

### IgG4-related disease

**Figure 1.**
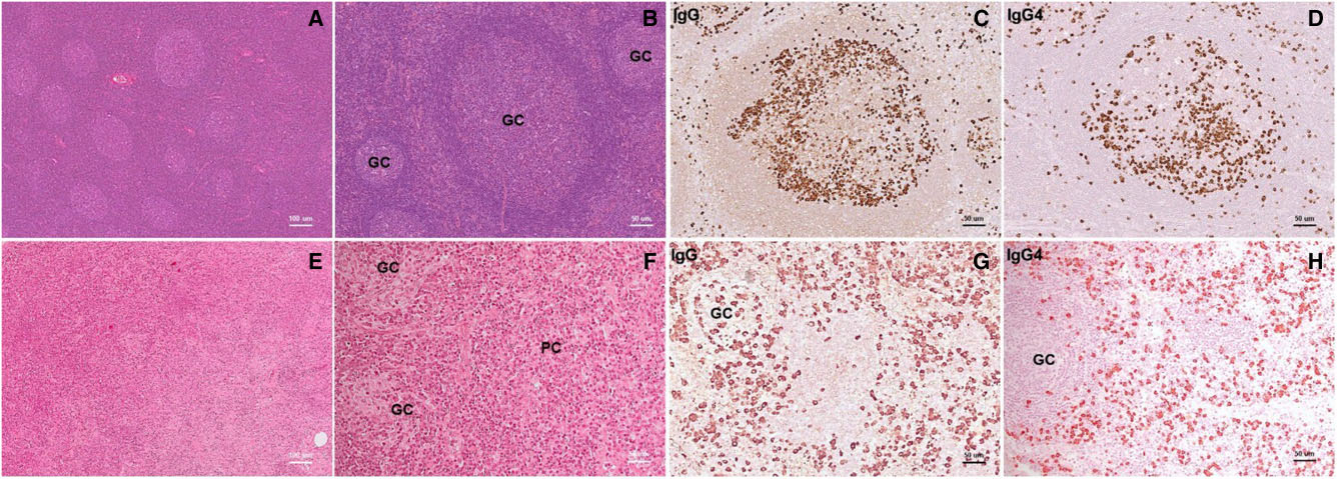
Morphological patterns in IgG4-related lymphadenopaty. (**A**–**D**) Follicular hyperplasia pattern, with preserved nodal architecture and IgG4+ plasma cells mainly distributed in germinal centres (GC); the IgG4+ to IgG+ ratio is >70%. (**E**–**H**) Castleman disease–like pattern with paracortical (PC) expansion and GCs showing regressive changes; IgG4+ plasma cells are mostly interfollicular and the IgG4+ to IgG+ ratio >70%. (A, B, E, F) Haematoxylin and eosin staining; (C, G) immunohistochemical staining for IgG; (D, H) immunohistochemical staining for IgG4

Lymphadenopathy is a common finding in the setting IgG4-RD, being reported in 30–55% of cases [51, 52]. Swollen lymph nodes are usually painless, with a diameter of 1–3 cm, though they can grow as large as 5 cm [53]. In this context, lymphadenopathy may become symptomatic due to compression of adjacent structures (e.g. hydronephrosis due to ureter compression, lower limbs oedema due to inferior vena cava or iliac vein compression). Lymphadenopathy is frequently generalized and the most commonly involved lymph node stations are mediastinal, axillary, intra-abdominal, cervical and inguinal [53]. Lymphadenopathy can be documented in IgG4-RD in four main clinical contexts. First, enlarged lymph nodes are incidentally found in the excision specimen of extranodal tissue demonstrating IgG4-RD features. Second, lymphadenopathy may be documented incidentally with physical examination or imaging during the diagnostic workup of extranodal disease. Third, lymphadenopathy may arise weeks to years after diagnosis of extranodal disease. Fourth, generalized lymphadenopathy may be the first presenting symptom of IgG4-RD, weeks to years before extranodal disease appears [10]. Recently, IgG4-RD has been arrayed into four clinical phenotypes, based on the observation of patterns of clinical presentation [54]. These groups are: (group 1) pancreato-hepatobiliary disease (31% of patients), (group 2) retroperitoneum and aorta (24%), (group 3) head and neck–limited (24%) and (group 4) Mikulicz’s syndrome with systemic involvement (22%). Lymphadenopathy is more frequently encountered in group 4, with 67% of frequency (second most common organ involved after submandibular gland). On the other hand, group 1 features the lowest frequency with 15%, while group 2 and 3 display lymphadenopathy in roughly 25% of cases [54]. At the microscopic level, lymph nodes can express five types of histologic pattern, all characterized by increased accumulation of IgG4+ plasma cells and by a IgG4/IgG ratio >40% at immunohistochemistry [55]. Pattern 1 is highly reminiscent of the plasmacytic histopathological subtype of MCD and is characterized by intact nodal architecture with hyperplastic follicles as well as follicles with some degree of germinal centre regression and possible penetration by venules. The interfollicular space displays increased high endothelial venules and abundant plasma cells. Pattern 2 shows a typical reactive follicular hyperplasia, encountered in other autoimmune diseases such as SLE and RA. Pattern 3 is characterized by normal or variably regressed follicles with marked expansion of the interfollicular space. This area is filled by prominent high endothelial venules, immunoblasts, small lymphocytes plasma cells and eosinophils. Pattern 4 is notable for the presence of ‘transformed’ germinal centres, two to four times larger than those of background follicles, which become ameboid or stellate shaped. Their follicles display a thickened mantle zone with inward protrusions into germinal centres. Pattern 5, known as ‘inflammatory pseudotumor-like’, is the least common and is characterized by replacement of the nodal parenchyma by sclero-hyaline tissue. Collagen fibers in this context may organize into a storiform pattern similar to that observed in other organ involved by IgG4-RD [10]. Importantly, histological patterns may not be fully discriminating and specimens may contain more than one pattern at a time (Fig. 1).

18F-FDG-PET enables clinicians to stage organ involvement, to select an amenable biopsy site, and to monitor response to treatment. However, PET is not able to distinguish IgG4-related lymphadenopathy from reactive lymphadenopathy [56].

The presence of IgG4-positive plasma cell infiltration in lymph nodes is not specific for IgG4-RD. In fact, isolated lymph nodes with IgG4-positive plasma cell infiltration and a compatible histological pattern is not enough to establish a diagnosis of IgG4-RD, even after exclusion of potential disease mimickers, as presence of extranodal disease is required [10]. The diagnosis of IgG4-RD requires a multimodality approach in which target organ involvement evaluated clinically and radiologically needs to be supported by laboratory and histological evidence as well as by exclusion of alternative diagnoses [57]. Differential diagnosis of IgG4-related lymphadenopathy is broad and mainly includes lymphoproliferative disorders, MCD, Ebstein–Barr related lymphadenopathy, sarcoidosis, Rosai Dorfman disease and unspecific reactive lymphadenopathy [10]. Patients with IgG4-positive plasma cell infiltration in lymph nodes need to be followed up strictly as they may develop extranodal involvement and be most appropriately diagnosed with IgG4-RD [10]. Lymphadenopathy has been recently described as a clinical marker of poor prognosis, associated with higher disease activity relapse rate [52].

### Castleman disease

First described in 1954 by Benjamin Castleman [58], CD is presently classified into unicentric (UCD), which involves a single region of enlarged lymph nodes, and multicentric (MCD), which involves multiple regions of enlarged lymph nodes and systemic inflammation. MCD is further classified based on the underlying aetiology into human herpesvirus 8–associated MCD (HHV8-MCD), POEMS (polyneuropathy, organomegaly, endocrinopathy, monoclonal plasma cell disorder, skin changes)-associated MCD (POEMS-MCD) and idiopathic MCD (iMCD), which has an unknown aetiology. Two types of iMCD are further distinguished: iMCD-TAFRO (thrombocytosis, ascites, reticulin fibrosis, renal dysfunction, organomegaly) and iMCD-NOS (not otherwise specified) [59].

CD is a rare disease, with an estimated annual incidence in the range of 15–20 cases per million patient years [60]. While no risk factors exist for the vast majority of CD patients, HIV infection predisposes patients to HHV8-associated MCD [61]. CD can occur in patients of all ages, including elderly and children, and tends to be evenly distributed throughout the lifespan [62–64].

UCD is thought to arise from clonal proliferation, most likely of lymph node stromal cells [65]. On the other hand, MCD involves excessive production of cytokines leading to a cytokine storm often including IL-6. Specifically, in HHV8-MCD, uncontrolled replication of HHV8 in plasmablasts of immunocompromised individuals results in aberrant production of the viral homologue of human IL-6 (vIL-6), which drives lymph node pathology as well as downstream transcription of several human proinflammatory cytokines [66, 67]. POEMS-MCD is likely driven by monoclonal plasma cells that after pathological gene translocations or deletions [67] produce a variety of cytokines including VEGF, IL-12 and IL-6, which have been proposed as the drivers of the clinical phenotype of POEMS-MCD [67, 68]. The aetiology of iMCD is presently unknown and probably subtends different processes resulting in immune dysregulation and increased secretion of proinflammatory cytokines such as VEFG and IL-6 [67].

#### Unicentric CD

UCD is characterized by swelling of one or multiple lymph nodes in a single station and is typically detected as an incidental finding during routine radiological exams or because of symptoms generated by compression of local anatomical structures [69]. Lymph nodes are often significantly enlarged (2–4 cm, up to 6+ cm) and, although UCD can occur in any lymph node station, the most commonly involved regions include the mediastinum, the abdomen and the cervical region [70]. The majority of patients are asymptomatic or present with symptoms related to mass effect; however, a minority may present with systemic inflammatory symptoms or develop systemic complications such as paraneoplastic pemphigus, bronchiolitis obliterans, polyneuropathy or haemolytic anaemia, some of which can be potentially fatal [70].

#### Multicentric CD

MCD involves lymphadenopathy in more than one node station, together with a plethora of systemic and organ-specific symptoms such as renal dysfunction, pulmonary involvement, liver dysfunction, fluid accumulation, cytopenia and constitutional symptoms [4, 70, 71]. At diagnosis, testing for HIV and HHV8 is mandatory to discriminate between HHV8-MCD, which is often HIV positive, and iMCD, which is HHV8- and HIV-negative. Identification of HHV8 in serum at PCR or positive testing of HHV8 latency-associated nuclear antigen (LANA-1) on lymph node biopsy allows this distinction to be made [71]. HHV8-MCD presents more frequently with haemophagocytic syndrome and Kaposi sarcoma [70]. HIV positivity has been associated with a higher frequency of fever, splenomegaly and haemophagocytosis [70]. Diagnosis of POEMS-MCD requires diagnosis of POEMS syndrome with concomitant identification of histology compatible with CD at lymph node biopsy. Classical manifestations are included in the POEMS acronym; to formulate a diagnosis of POEMS, polyneuropathy and monoclonal plasma cell disorder (almost always λ) need to be present, with either lymph nodes compatible with CD, presence of sclerotic bone lesions or elevation of VEGF [72]. iMCD more frequently presents fluid accumulation and renal dysfunction [73]. There is a broad range of phenotypes within iMCD, including a distinctive clinical phenotype characterized by thrombocytopenia, ascites, reticulin fibrosis, renal dysfunction and organomegaly (iMCD-TAFRO), which represents the most aggressive subtype of CD [74]. The proposed diagnostic criteria for iMCD-TAFRO require demonstration of HHV8-negative-MCD and at least three out of fever, thrombocytopenia, anasarca, reticulin fibrosis and organomegaly, plus at least one of megakaryocyte hyper/normoplasia at bone marrow biopsy or high alkaline phosphatase without marked transaminase elevation [74]. Other patients present with thrombocytosis, hypergammaglobulinemia and a less severe clinical presentation, which are often called idiopathic plasmacytic lymphadenopathy (iMCD-IPL) subtype or not otherwise specified (iMCD-NOS).

#### Castleman disease histology

**Figure 2.**
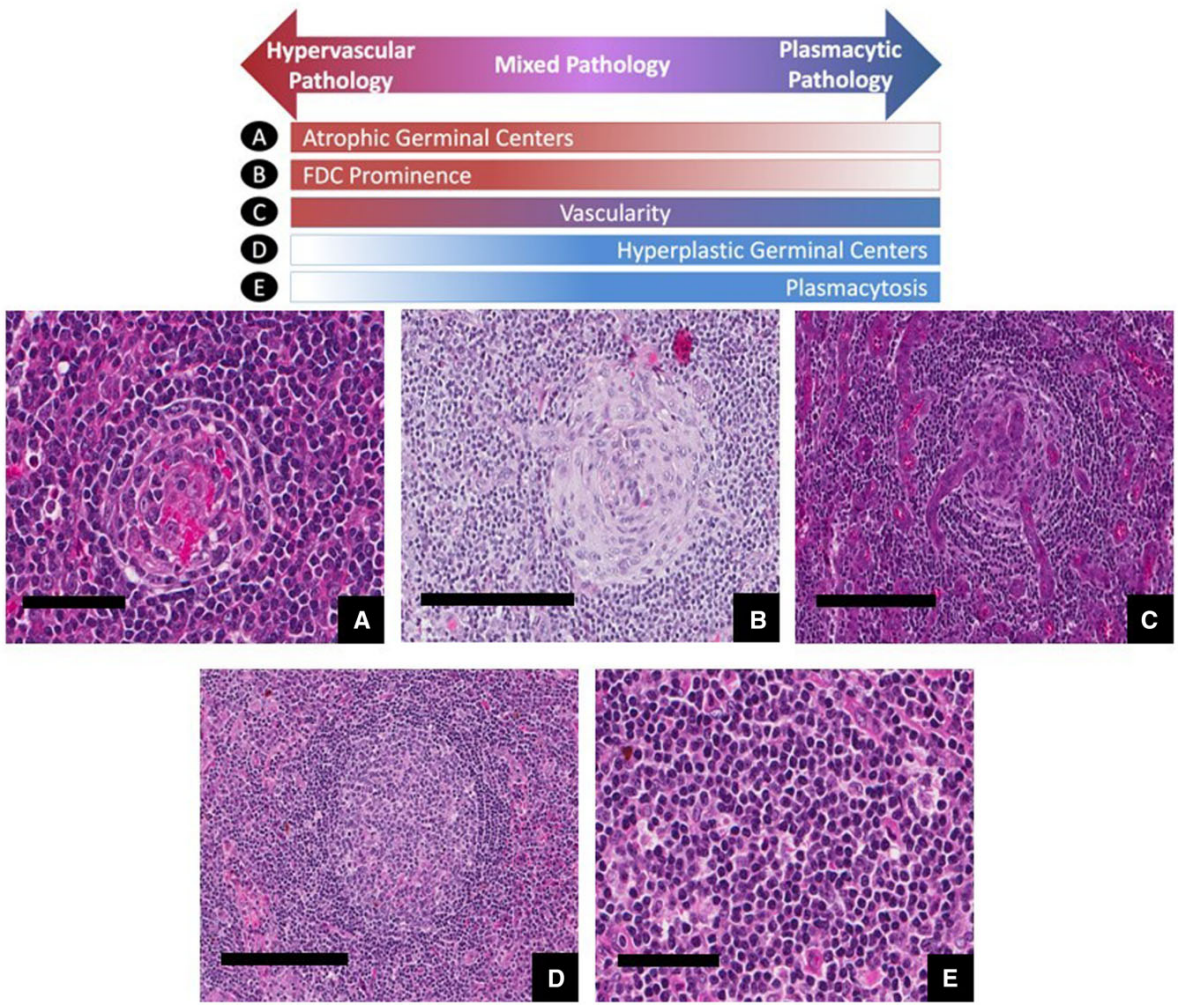
Histological feature and patterns in Castleman disease. This research was originally published in *Blood* (Fajgenbaum DC *et al*. International, evidence-based consensus diagnostic criteria for HHV8–negative/idiopathic multicentric Castleman disease. *Blood* 2017;129:1646–57 [59]). © The American Society of Hematology. Hypervascular subtype is characterized by the presence of atretic germinal centres (**A**) predominantly composed by follicular dendritic cells (FDC) (**B**), whereas the plasmacytic subtype exhibits hyperplastic germinal centres (**D**) and profuse sheetlike plasmacytosis (**E**). Mixed subtype exhibits a combination of hypervascular and plasmacytic features. Prominent vascularity with blood vessels penetrating germinal centres (**C**) is frequently observed in iMCD, but can be seen with either subtype. Deidentified lymph node slides were obtained prestained with haematoxylin and eosin from Janssen Pharmaceuticals and scanned using an Aperio CS scanner (Leica Biosystems, Wetzlar, Germany) at 20×/0.75NA Plan Apochromat. Images were captured using an Aperio Imagescope and enhanced to 300 d.p.i. using Adobe Photoshop. Bars represent 60 μm (A, E), 200 μm (B–D). iMCD: idiopathic multicentric Castleman disease

Three histological subtypes of CD have been described but it is important to note that the features occur across a spectrum and it is very difficult to distinguish between them: (i) the hyaline vascular (UCD) or hypervascular (MCD) pattern; (ii) the plasmacytic pattern; and (iii) a mixed pattern of both. Hyaline vascular pattern is characterized by capsular fibrosis with radially penetrating sclerotic vasculature and obliteration of lymph node sinuses; this is only found in UCD. The hyaline vascular pattern is observed in roughly 90% of cases of UCD [64]. The term ‘hypervascular’ is used to refer to these histological characteristics in iMCD, with the notable difference that usually the lymph node architecture is preserved and sinuses are not obliterated [75]. In the hyaline vascular or hypervascular histopathological subtypes, follicles are atretic with a reduced number of lymphocytes and predominantly composed of follicular dendritic cells, while the surrounding mantle is hyperplastic, often containing more than one germinal centre, and there is usually a marked vascular proliferation with prominent endothelial cells in the interfollicular zones [75]. Hypervascular histopathology is frequently documented among iMCD-TAFRO cases [74]. Plasmacytic pattern is characterized by the presence of polyclonal plasma cells, often organized in sheets, in the interfollicular zone. Lymph node architecture is usually preserved and shows features of reactive follicular hyperplasia with hyperplastic germinal centres. This pattern is more frequently observed in HHV8-MCD, POEMS-MCD, and iMCD-IPL or iMCD-NOS, while it is rarely reported in UCD or iMCD-TAFRO [75]. Cases of HHV8-MCD are notable for presence of interfollicular plasmablasts positive for HHV8 LANA-1 and often vIL-6 [75]. These cells are typically polyclonal but may seldom be monoclonal and progress towards lymphoma, currently termed HHV8-positive diffuse large B-cell lymphoma [76]. Mixed pattern is used to describe patients with both hyaline vascular/hypervascular and plasmacytic features, with regressed germinal centres and interfollicular sheets of plasma cells. It may be observed in iMCD or UCD [64]. Fig. 2 depicts the different histological features and patterns that may be found in CD.

#### Castleman disease treatment

The treatment of UCD consists of surgical removal whenever possible, which is typically curative. Extranodal symptoms, when present, usually regress within 12 months, with the possible exception of bronchiolitis obliterans [7, 64, 77]. Rare cases of recurrence after surgery have been reported [77]. When surgery is not feasible, irradiation, embolization or immunosuppressive therapy with IL-6 blocking agents can be considered [64]. Treatment of MCD requires different therapy based on the underlying aetiology. Pharmacological treatment of HHV8-MCD is largely based on rituximab therapy to eliminate the CD20-positive plasmablasts, with the addition of etoposide in high-risk patients (i.e. with poor performance status, haemophagocytic syndrome, target organ insufficiency) [78, 79]. This is associated with a 95% of achievement of clinical remission, with 92% 5-year overall survival and 82% relapse-free survival [79]. Antiretroviral therapy should be added in case of HIV positivity. Treatment of POEMS-MCD is directed at the monoclonal plasma cells and similar to multiple myeloma, with high-dose chemotherapy and autologous stem cell transplantation. In rare cases of POEMS-MCD without osteosclerotic bone lesions, therapy with anti-IL-6 agents might be considered [64].

The treatment of iMCD has significantly changed, with the introduction of IL-6 blocking therapy now representing the first-line therapy according to international guidelines [80]. In particular, siltuximab—an mAb targeting soluble IL-6—is the only drug tested in a randomized clinical trial and the only one approved by the Food and Drug Administration and by the European Medicines Agency for iMCD [81]. In *post hoc* and retrospective analyses, siltuximab proved superior in terms of progression-free survival when compared with placebo and with rituximab and chemotherapy-based regimens [82, 83]. If siltuximab is not available, tocilizumab, an mAb against soluble and membrane-bound IL-6 receptor, may be employed off-label [84]. Still, ∼50–66% of patients do not show a significant response to IL-6 inhibition [81]. These patients typically show elevation of biomarkers associated with IL-6-driven inflammation such as IL-6 itself, fibrinogen, CRP, total serum IgG, triglycerides and erythropoietin [85–87]. Cessation of IL-6 blockade frequently causes disease relapse, therefore lifelong therapy is recommended, with the possibility of extending the interval between doses in patients stably on remission [80]. Rituximab as well as immunosuppressive/immunomodulatory agents such as ciclosporin A, sirolimus, thalidomide and anakinra may be employed as an alternative to anti-IL-6 agents or as second-line therapy in non-severe iMCD cases [80]. Overall, these regimens are associated with an ∼35–50% rate of response but relapses are frequent [64, 80, 88]. In severe cases, such as iMCD-TAFRO, anti-IL6 therapy in association with high dose glucocorticoids still represents the treatment of choice but multi-agent chemotherapy should be reconsidered in case of non-response [80].

### Other diseases

**Figure 3.**
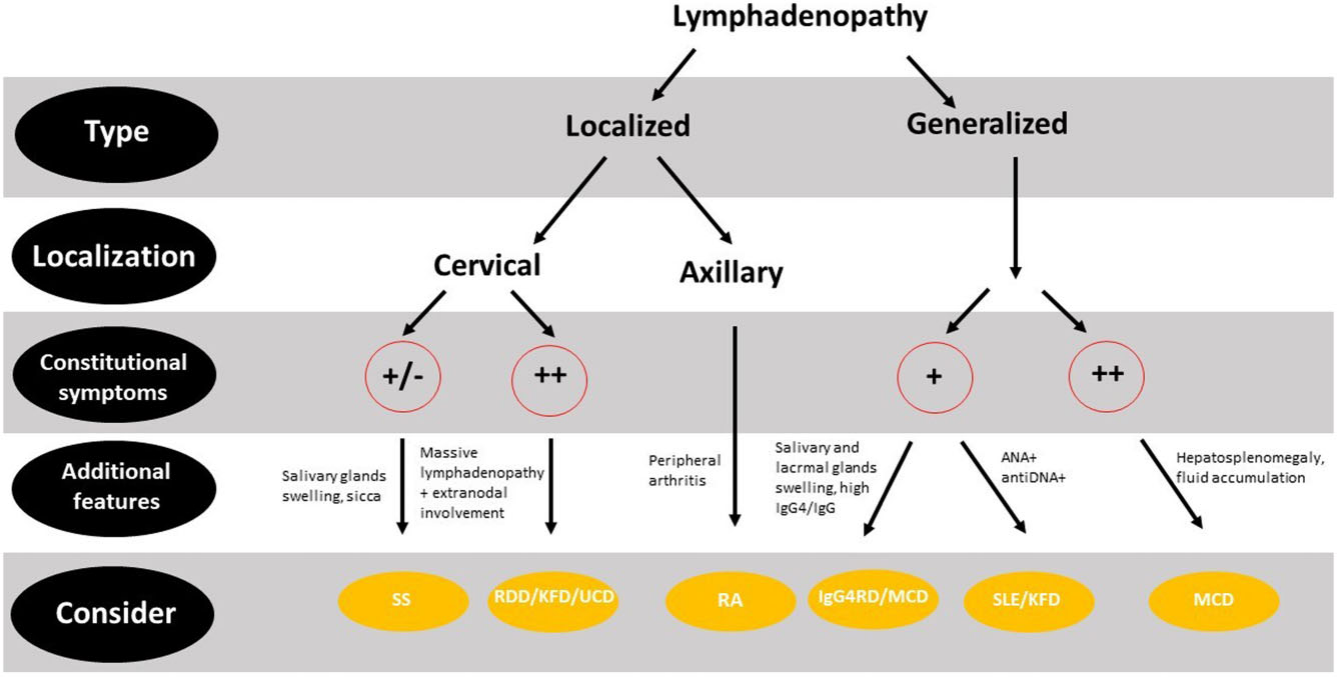
Flowchart for interpretation of lymphadenopathy in rheumatological diseases. Lymphadenopathy should be long-lasting and differential diagnosis with infectious and neoplastic aetiologies should always be exhaustive. KFD: Kikuchi Fujimoto disease; RDD: Rosai Dorfman disease; IgG4RD: IgG4-related disease; UCD: unicentric Castleman disease; MCD: multicentric Castleman disease

Rare rheumatological diseases worth mentioning due to their peculiar lymph node involvement are Kikuchi Fujimoto disease, a rare necrotizing lymphadenitis with possible life-threatening manifestations (e.g. aseptic meningitis, myocarditis, haemophagocytic syndrome), and Rosai Dorfman disease, a rare non-Langherans cell histiocytosis characterized by massive lymphadenopathy and systemic and organ-specific symptoms. These diseases will not be covered in this review but have been excellently reviewed elsewhere [89, 90]. Fig. 3 depicts a simplified flowchart for the interpretation of lymphadenopathy in the various rheumatological diseases reviewed here.

## Conclusions

Lymphadenopathy is a frequent clinical finding in rheumatology practice and can often be interpreted as a clinical marker of disease activity. Moreover, lymphadenopathy can be the initial or more prominent manifestation of rare rheumatologic diseases, such as IgG4-RD and CD, the complications of which are potentially life-threatening. Early recognition and prompt treatment of these diseases may significantly impact on mortality and morbidity. Nevertheless, since rheumatologic patients are at higher risk of infectious and neoplastic complications, a careful differential diagnosis is of the utmost importance.

## Data Availability

No new data were generated or analysed in support of this research.
